# Substrate-analogue complex structure of *Mycobacterium tuberculosis* decaprenyl diphosphate synthase

**DOI:** 10.1107/S2053230X19001213

**Published:** 2019-03-13

**Authors:** Tzu-Ping Ko, Xiansha Xiao, Rey-Ting Guo, Jian-Wen Huang, Weidong Liu, Chun-Chi Chen

**Affiliations:** a Institute of Biological Chemistry, Academia Sinica, Taipei 11529, Taiwan; b Industrial Enzymes National Engineering Laboratory, Tianjin Institute of Industrial Biotechnology, Chinese Academy of Sciences, Tianjin 300308, People’s Republic of China; c State Key Laboratory of Biocatalysis and Enzyme Engineering, Hubei Collaborative Innovation Center for Green Transformation of Bio-Resources, Hubei Key Laboratory of Industrial Biotechnology, School of Life Sciences, Hubei University, Wuhan 43420, People’s Republic of China

**Keywords:** Rv2361c, *cis*-prenyltransferase, catalytic mechanism, thiodiphosphate, inhibitor, cell-wall biosynthesis, *Mycobacterium tuberculosis*, decaprenyl diphosphate synthase

## Abstract

Decaprenyl diphosphate synthase from *Mycobacterium tuberculosis* (*Mt*DPPS, also known as Rv2361c) catalyzes the consecutive elongation of ω,*E*,*Z*-farnesyl diphosphate (*EZ*-FPP) by seven isoprene units by forming new *cis* double bonds. The protein folds into a butterfly-like homodimer like most other *cis*-type prenyltransferases. The starting allylic substrate *EZ*-FPP is bound to the S1 site and the homoallylic substrate to be incorporated, isopentenyl diphosphate, is bound to the S2 site. Here, a 1.55 Å resolution structure of *Mt*DPPS in complex with the substrate analogues geranyl *S*-thiodiphosphate (GSPP) and isopentenyl *S*-thiodiphosphate bound to their respective sites in one subunit clearly shows the active-site configuration and the magnesium-coordinated geometry for catalysis. The ligand-binding mode of GSPP in the other subunit indicates a possible pathway of product translocation from the S2 site to the S1 site, as required for the next step of the reaction. The preferred binding of negatively charged effectors to the S1 site also suggests a promising direction for inhibitor design.

## Abstract 2

The structure of a complex of *Mycobacterium tuberculosis* decaprenyl diphosphate synthase with geranyl *S*-thiodiphosphate and isopentenyl *S*-thiodiphosphate was refined to 1.55 Å resolution. It not only shows the magnesium-coordinated configuration for catalysis but also suggests a pathway for product translocation as well as a direction for inhibitor design.

## 1. Introduction   

Tuberculosis (TB), which is one of the top ten causes of death worldwide, is a long co-existing human ‘crowd’ disease that is caused by a group of genetically related mycobacteria, namely the *Mycobacterium tuberculosis* complex (MTBC). The emergence of multidrug-resistant TB (MDR-TB) is a major threat to public health, and the goal of eliminating this disease in the near future is being challenged (World Health Organ­ization, 2018 ▸). In the development of new therapeutics, targeting the structurally similar enzymes Rv1086, Rv2361c and Rv3378c that are involved in cell-wall biosynthesis and virulence-factor production may be a possible approach (Wang *et al.*, 2008 ▸; Chan *et al.*, 2014 ▸). All three proteins belong to the *cis*-type prenyltransferase (*cis*-PT) family, which adopt the ζ-fold (Oldfield & Lin, 2012 ▸). Rv1086 produces ω,*E*,*Z*-farnesyl diphosphate (*EZ*-FPP; C_15_) from geranyl diphosphate (GPP; C_10_) and isopentenyl diphosphate (IPP; C_5_), which is used by Rv2361c for further elongation to form decaprenyl diphos­phate (DPP; C_50_; Kaur *et al.*, 2004 ▸). Rv3378c transfers the diterpene moiety of tuberculosinyl diphosphate (TPP; C_20_) to adenosine, turning out 1-tuberculosinyladenosine (1-TbAd; Layre *et al.*, 2014 ▸) as an abundant MTBC marker.

The reaction catalyzed by Rv2361c (or *M. tuberculosis* DPP synthase, *Mt*DPPS) is very similar to that of undecaprenyl diphosphate synthase (UPPS; see, for example, Ko *et al.*, 2018 ▸), except for the chain length of the final product (C_50_ versus C_55_) and the starting allylic substrate (*EZ*-FPP versus *EE*-FPP). In fact, most *cis*-PTs share a common dimeric architecture, and the conserved S1 and S2 sites for substrate binding are located near the subunit interface (Oldfield & Lin, 2012 ▸). The starting allylic substrate is bound to the S1 site and the homoallylic substrate to be incorporated is bound to the S2 site. An invariant aspartate residue plays a central role in catalysis by coordinating the Mg^2+^-bound substrates (Guo *et al.*, 2005 ▸). Other conserved residues, including the Asn–Ser proton shuttle and the C-terminal R*X*G motif, are also catalytically important (Malwal *et al.*, 2018 ▸; Grabińska *et al.*, 2017 ▸; Chan *et al.*, 2017 ▸). The head-to-tail coupling reaction of *cis*-PT proceeds through a concerted pathway similar to the ionization–condensation–elimination mechanism of *trans*-PT (Liang, 2009 ▸). After the formation of the new C—C bond, the pyrophosphate leaves the S1 site along with Mg^2+^, and the resulting prenyl diphosphate switches from the S2 site to the S1 site (Guo *et al.*, 2005 ▸; Supplementary Fig. S1).

The crystal structure of *Mt*DPPS was first determined in a trigonal unit cell at 1.8–2.6 Å resolution, which revealed an N-terminal module besides the Rossmann-fold-like domain of *cis*-PT (Wang *et al.*, 2008 ▸; PDB entries 2vg2, 2vg3 and 2vg4). In one crystal the S1 site contained the substrate analogue citronellyl diphosphate (CitPP; C_10_), which mimics GPP in structure but lacks the allylic double bond, while in another the S2 site contained an IPP molecule (Supplementary Fig. S2). Subsequently, the structure of the *Mt*DPPS–bisphosphon­ate complex with BPH-640 was solved using an ortho­rhombic crystal (Chan *et al.*, 2014 ▸; PDB entry 4onc), in which the inhibitor was bound to the S1 site (Supplementary Fig. S3*a*). Here, we report a complex structure at 1.55 Å resolution with both the S1 and S2 sites occupied by the respective allylic and homoallylic substrate analogues. Some features that provide additional insights into the catalytic mechanism are discussed.

## 2. Materials and methods   

### 2.1. Production and purification of *Mt*DPPS   

Recombinant *Mt*DPPS (Rv2361c) protein was expressed in *Escherichia coli* using the vector pET-28a and was purified as described previously (Wang *et al.*, 2008 ▸). Briefly, His-tagged *Mt*DPPS was loaded onto an Ni–NTA column (Qiagen), washed and eluted with an imidazole gradient. The His tag was removed by thrombin cleavage and cation exchange using a POROS HS20 column (ThermoFisher). The protein was further purified by gel filtration using a HiPrep 16/60 S-100 column (GE Healthcare) and was concentrated to 10 mg ml^−1^ in 20 m*M* Tris pH 7.2, 0.15 m*M* MgCl_2_, 3 m*M* dithiothreitol for crystallization.

### 2.2. Crystallization, data collection and structure determination   

Following previously described procedures (Chan *et al.*, 2014 ▸), recombinant *Mt*DPPS was crystallized by the sitting-drop vapour-diffusion method at 25°C using a reservoir solution consisting of 0.1 *M* HEPES pH 7.5, 10% glycerol, 25% PEG 400, 7% PEG 3000. Prior to data collection, the crystals were soaked in reservoir solution with 1 m*M* each of isopentenyl *S*-thiodiphosphate (ISPP; C_5_) and geranyl *S*-thiodiphosphate (GSPP; C_10_) for 6 h.

X-ray diffraction data were collected from the *Mt*DPPS crystal on beamline BL15A1 at the National Synchrotron Radiation Research Center (NSRRC) and were processed using *HKL*-2000 (Otwinowski & Minor, 1997 ▸). The ortho­rhombic crystal has the same space group as, and similar unit-cell dimensions to, PDB entry 4onc (Chan *et al.*, 2014 ▸). Further structural refinement was carried out with *PHENIX* (Adams *et al.*, 2010 ▸) and *Coot* (Emsley & Cowtan, 2004 ▸). Some statistics for the data and model are listed in Table 1 ▸.

## 3. Results   

### 3.1. Overall structure of *Mt*DPPS   

The initial *R* value of 0.242 obtained after rigid-body refinement at 2.5 Å resolution confirmed that the crystal structure was isomorphous to that of PDB entry 4onc, with a dimer of *Mt*DPPS in the asymmetric unit. However, significant structural rearrangements were observed in the active-site region, adjacent to Asp76, in both subunits. One active site contained GSPP, ISPP and Mg^2+^, while the other only contained GSPP. Probably owing to the presence of bound substrate analogues, a previously disordered C-terminal segment became visible (Supplementary Fig. S3*b*). In addition to incorporating these and other ligands, as well as solvent molecules, the use of local NCS restraints and TLS parameters in the refinement greatly improved the model as evaluated by *MolProbity* (Chen *et al.*, 2010 ▸; Table 1 ▸). The refined model to 1.55 Å resolution differs from the original model by an r.m.s.d. of 0.167 Å for 482 pairs of matched C^α^ atoms, which is even lower than the 0.258 Å r.m.s.d. between the two subunits for 246 C^α^ pairs. The largest deviations occur at the C-terminus, which contains the R*X*G motif. Comparison with 14 other known homologous structures indicates that the conformation is most similar to those of the closed UPPSs from *E. coli* and from *Staphylococcus aureus* (PDB entries 1x09 and 4h8e; Guo *et al.*, 2005 ▸; Zhu *et al.*, 2013 ▸), with r.m.s.d.s of 0.84–1.00 Å for 193–197 C^α^ pairs in the monomers.

**Figure 1:**
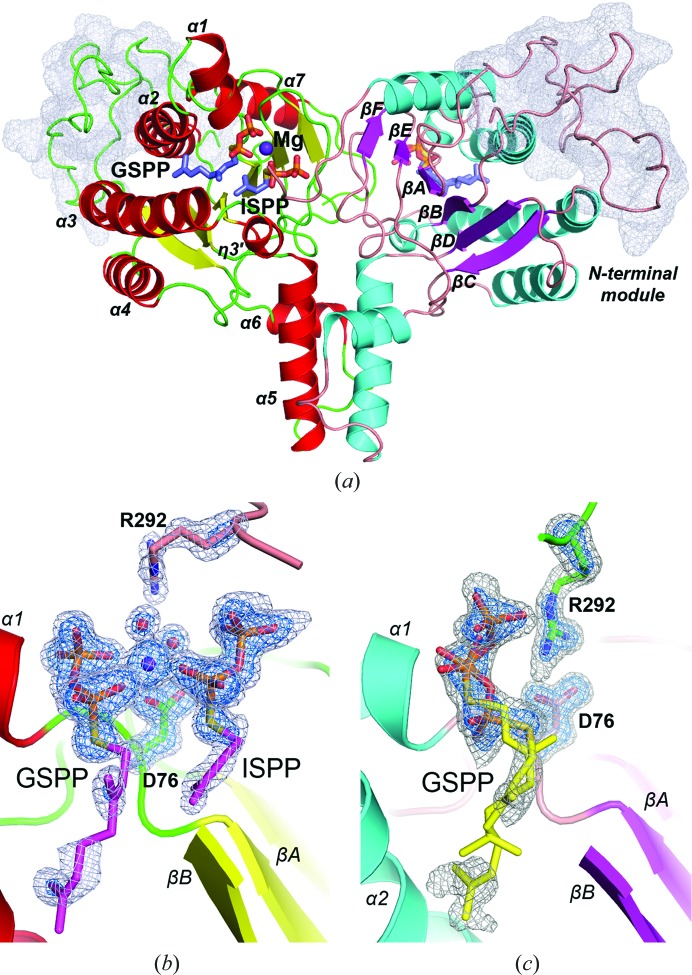
Overall structure of *Mt*DPPS. (*a*) The two monomers in the asymmetric unit of the *Mt*DPPS crystal are shown as ribbon diagrams. The β-strands are named A–F and the α-helices are numbered 1–7 from the N-terminus to the C-terminus. They are coloured yellow/red for one subunit and magenta/cyan for the other subunit. The surface of the N-terminal module is shown as a grey mesh. (*b*) The *F*_o_ − *F*_c_ map calculated by omitting the bound GSPP, ISPP (magenta sticks) and Mg^2+^ (purple sphere) as well as the associated water molecules (red spheres) and the side chains of Asp76 and Arg292 (green and orange sticks) is contoured at 3σ and 5σ levels and shown as grey and blue mesh, respectively. (*c*) The *F*_o_ − *F*_c_ map calculated by omitting the bound GSPP (yellow sticks) and the side chains of Asp76 and Arg292 (orange and green sticks) is contoured at 3σ and 5σ.

*Mt*DPPS contains a unique N-terminal module that is not seen in any other known *cis*-PT (Wang *et al.*, 2008 ▸; Fig. 1 ▸ *a*). It is located on the far side of the dimer. There are 15 prolines in the 50-residue cap-like moiety. A search with *DALI* (Holm & Laakso, 2016 ▸) suggested that the fold of this N-terminal cap is unique. No canonical secondary structure is present despite a few backbone hydrogen bonds, but the L-shaped protein fold is reminiscent of the tRNA structure (Supplementary Fig. S4). It interacts mainly with the α2, α3 and α7 helices (Fig. 1 ▸ *a*), burying a surface area of 1200 Å^2^ and involving more than 20 amino acids on each side. The interface comprises both non­­polar and polar residues, among which the most prominent are Phe26, Trp32, Phe36 and His53 on one side and Glu109, Arg146, Leu153 and Trp280 on the other side (Supplementary Fig. S5). Helices α2 and α3 constitute part of the tunnel that holds the hydrocarbon tail of the product. In other *cis*-PTs such as *E. coli* UPPS they are directly exposed to solvent and undergo an open–closed conformational change to facilitate catalysis. In comparison, *Mt*DPPS adopts only the closed conformation, presumably owing to the presence of the N-terminal cap.

### 3.2. Substrate-binding modes   

Unlike the previous *Mt*DPPS structures, which had substrate or inhibitor molecules bound to either the S1 site or the S2 site, the refined structure at 1.55 Å resolution in this study contains both the S1 and S2 ligands as well as the bound metal ion (Fig. 1 ▸ *b*). Although the hydrocarbon moiety of GSPP is somewhat disordered in the tunnel, presumably owing to the *cis*/*trans* difference in the allylic double bond from the real substrate, other parts such as the positions of the S and C1 atoms, where the starting ionization reaction occurs, can be seen unambiguously. The nucleophilic C4 atom of ISPP is almost collinear with the C1—S bond of GSPP, at a distance of 3.5 Å. With the *cis*-like positioning of the C1 and C4 atoms, the homoallylic substrate is ready to attack the allylic substrate and form a new double bond in this configuration. The Mg^2+^ ion forms an octahedral coordination with both phosphate groups of GSPP, the α-phosphate of ISPP, the side chain of Asp76 and two water molecules. One water is hydrogen-bonded to the β-phosphate of ISPP and the side chains of Asp76 and Arg292*. The other is hydrogen-bonded to the GSPP β-phosphate and the Arg292* side chain. (An asterisk denotes a residue from the counter-subunit in a dimer.)

The bound GSPP in the other active site shows two alternate conformations with similar occupancies and temperature factors (Fig. 1 ▸ *c*). One assumes the same position as that in the other subunit, despite some rotation and shift of the α-phosphate and the allylic prenyl group. The other has the entire molecule moved outwards, with its β-phosphate facing the solvent, its α-phosphate in the position of the β-phosphate of the first conformer and its prenyl group halfway into the tunnel. This active site contains neither an S2 ligand nor an Mg^2+^ ion, but the negatively charged side chain of Asp76 forms two direct hydrogen bonds to the positively charged Arg292*, which also binds to the β-phosphate in the second conformer of GSPP. Interestingly, the thiodiphosphate group of this conformer correlates well with the head group of the bisphosphonate inhibitor BPH-640 (Chan *et al.*, 2014 ▸; Supplementary Fig. S6), suggesting that the S1 site contains a third subsite for phosphate and may be capable of triphos­phate binding. In the recently determined *Acinetobacter* UPPS structure, a citrate was also bound to a similar location adjacent to the S1 site (Ko *et al.*, 2018 ▸; Supplementary Fig. S6).

## 4. Discussion   

With the exception of rubber synthase, which makes long-chain products without a particular size limit, most other *cis*-PTs (and also *trans*-PTs) accommodate the elongating chain in a nonpolar pocket surrounded by hydrophobic side chains from helices α2/α3 and the central β-sheet (or from the four core α-helices in *trans*-PTs; Liang *et al.*, 2002 ▸). The size of this pocket determines the product chain length. As a processive enzyme, *Mt*DPPS does not release its product, which is to become the allylic substrate in the next cycle of catalysis, until a final chain length of C_50_ is reached. Presumably, the hydrocarbon-tail moiety of the allylic substrate or product undergoes a certain rearrangement in the pocket, but it must be accompanied by translocation of the pyrophosphate moiety from the S2 site to the S1 site after each cycle of the elongation reaction. The alternative binding modes of GSPP in the magnesium-free subunit of *Mt*DPPS, which are suggestive of a third phosphate subsite, may represent the transition state of such a translocation.

**Figure 2:**
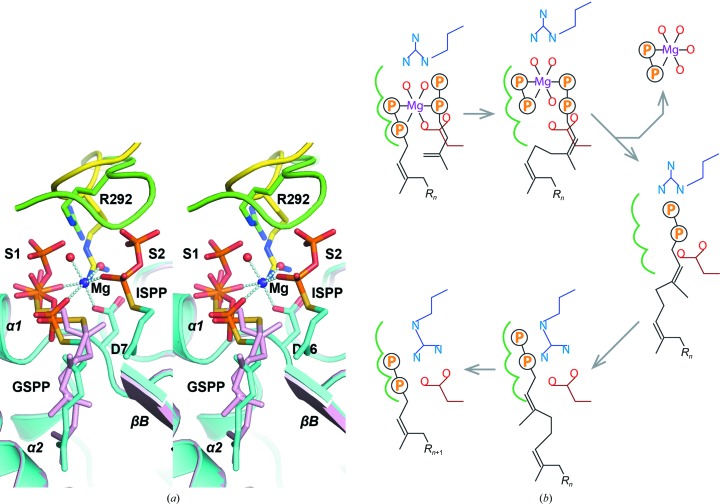
Binding modes of substrate analogues. (*a*) In this stereo diagram, the *Mt*DPPS dimer is superimposed on itself with the two polypeptide chains switched. The protein is coloured cyan/green in one dimer and pink/yellow in the other, as are the side chains and the ligands, which are shown as stick models. Mg^2+^ ions and water molecules are shown as spheres, and the coordinate bonds are shown as strings of beads. The locations of the S1 and S2 sites as well as the nearby helices α1/α2 and strand βB are also indicated. (*b*) In this schematic diagram, the side chains of Asp76 and Arg292 are coloured dark red and dark blue, respectively. The three subsites for the alternative binding modes of the S1 substrate are indicated by green curves. Other bonds, including the magnesium coordination, are shown in black. *R*_*n*_ represents a group of *n* consecutive isoprene units (C_5*n*_).

As shown in Fig. 2 ▸(*a*), when the S1 and S2 substrates and Mg^2+^ are properly bound for catalysis, the side chain of Asp76 not only binds directly to Mg^2+^ but also to a coordinating water molecule. The same water is hydrogen-bonded to the side chain of Arg292*, which also binds to the other magnesium-bound water. In the absence of both the S2 substrate and Mg^2+^, Arg292* turns to bind directly to Asp76, which is no longer engaged in magnesium coordination (Fig. 2 ▸ *a*). The side chain of Arg292* binds to the β-phosphate of the S1 substrate in this conformation, and in the other conformation it is also close to the β-phosphate of the S2 substrate. Consequently, it appears to be the most likely candidate to serve as the carrier for translocating the S2 ligand to the S1 site by binding to the β-phosphate. As depicted in Fig. 2 ▸(*b*), after the formation of the new *cis* double bond the S1 pyrophosphate dissociates as a magnesium complex, and Arg292* binds to the β-phosphate of the product and transfers it to the S1 site. While the five-carbon-longer hydrocarbon tail needs structural rearrangements to fit into the S1 pocket, the diphosphate moiety may be disposed like those of the GSPP conformers before it assumes a productive binding mode for the next cycle of reaction.

In summary, the *Mt*DPPS structure presented here with the bound substrate analogues GSPP and ISPP clearly shows the active-site configuration and the magnesium-coordinated geometry for catalysis, consistent with previous crystallo­graphic observations and biochemical studies of *cis*-PTs in general. It also shows that the S1 site is likely to be a preferred binding site for ligands that contain multiple negatively charged groups, such as GSPP and BPH-640. GSPP shows alternative binding modes that are likely to be adopted by the reaction intermediate. The previously observed BPH-640 also has its bisphosphonate group in a similar location. Consequently, the S1 site could be a promising target for inhibitor design against *Mt*DPPS, which might start with triphosphate analogues that contain various hydrocarbon tails.

## Supplementary Material

PDB reference: Rv2361c, 6ime

Supplementary Figures.. DOI: 10.1107/S2053230X19001213/no5157sup1.pdf

## Figures and Tables

**Table 1:** Data-collection and structure-refinement statistics Values in parentheses are for the outermost resolution shell. All positive reflections were used in refinement.

PDB code	6ime
Data collection	
Beamline	BL15A, NSRRC
Wavelength (Å)	1.0000
Space group	*P*2_1_2_1_2_1_
*a*, *b*, *c* (Å)	77.71, 89.12, 94.44
Resolution range (Å)	25–1.55 (1.61–1.55)
Unique reflections	95419 (9410)
Multiplicity	6.3 (6.3)
Completeness (%)	99.9 (100.0)
Average *I*/σ(*I*)	48.7 (4.8)
Average CC_1/2_	0.996 (0.981)
*R*_merge_ (%)	3.6 (42.9)
*R*_p.i.m._ (%)	1.6 (18.6)
Structure refinement	
No. of reflections	95335 (9261)
Completeness (%)	99.7 (98.2)
*R*_work_ (95% of data)	0.145 (0.185)
*R*_free_ (5% of data)	0.167 (0.206)
R.m.s.d., bond lengths (Å)	0.0126
R.m.s.d., bond angles (°)	1.35
No. of atoms	
Protein	4688
Ligand	92
Water	752
Average *B* factor (Å^2^)	
Protein	24.0
Ligand	37.7
Water	40.3
Ramachandran favoured (%)	97.7
Ramachandran allowed (%)	2.3
Ramachandran outliers (%)	0.0
Rotamer outliers (%)	0.78
Clashscore	4.32
*MolProbity* score	1.28
