# Supplementary material for: Substrate-analogue complex structure of Mycobacterium tuberculosis decaprenyl diphosphate synthase
Source: Acta Crystallogr F Struct Biol Commun. 2019 Mar 13;75(Pt 4):212–6. doi: 10.1107/S2053230X19001213 (PMC6450523; doi:10.1107/S2053230X19001213)
Supplement: Supplementary file 1 [file f-75-00212-sup1.pdf]

STRUCTURAL BIOLOGY
COMMUNICATIONS

Volume 75 (2019)

Supporting information for article:

Substrate-analogue complex structure of *Mycobacterium tuberculosis* decaprenyl diphosphate synthase

Tzu-Ping Ko, Xiansha Xiao, Rey-Ting Guo, Jian-Wen Huang, Weidong Liu and Chun-Chi Chen

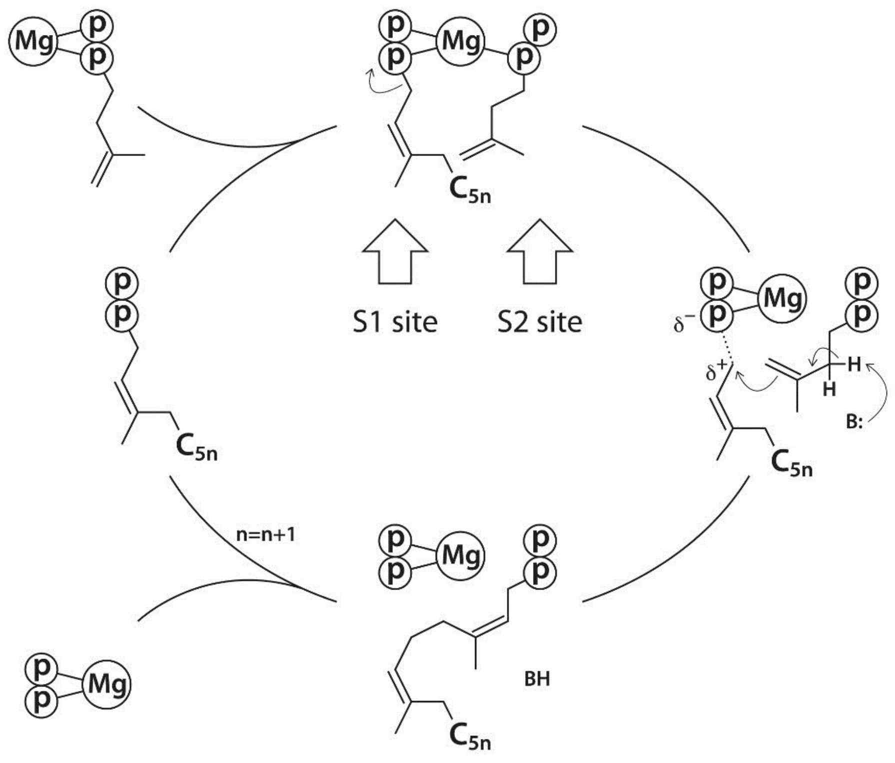

Figure S1 The reaction pathway of *cis*-prenyltransferase in general. C_{5n} stands for a hydrocarbon group of n prenyl units.

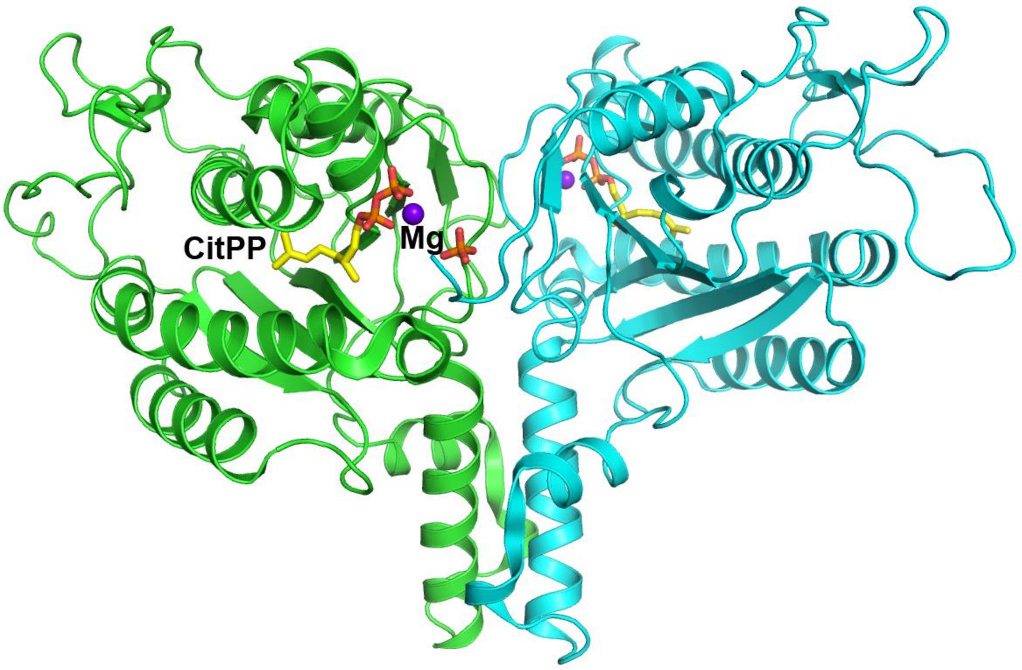

(*a*)

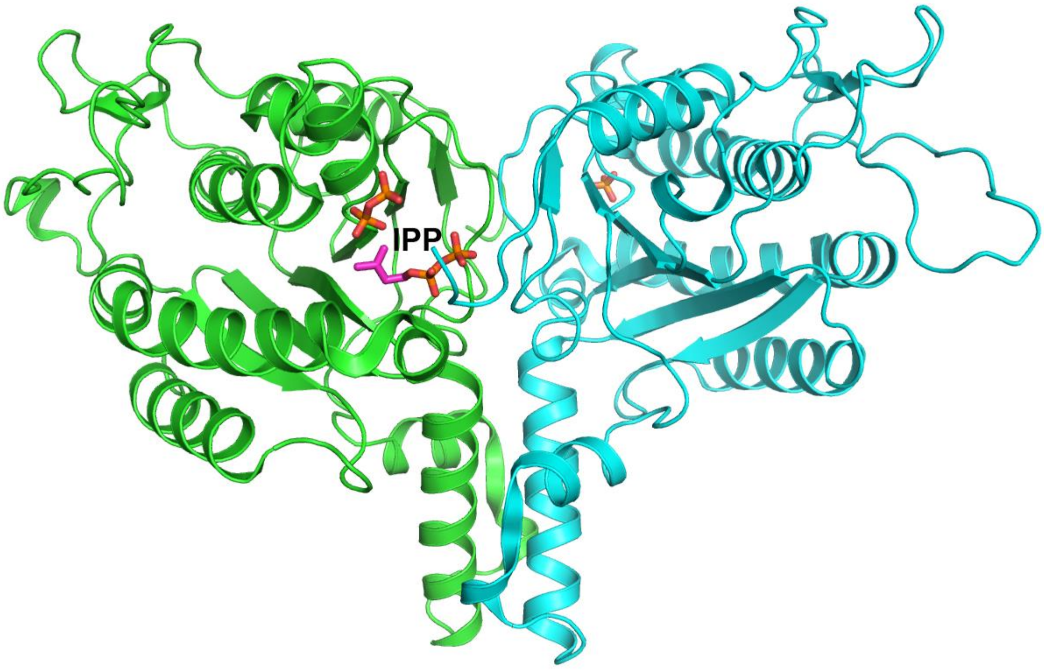

(*b*)

Figure S2 Supporting information The bound ligands to *Mt*DPPS in (*a*) PDB 2vg3 and (*b*) 2vg2.

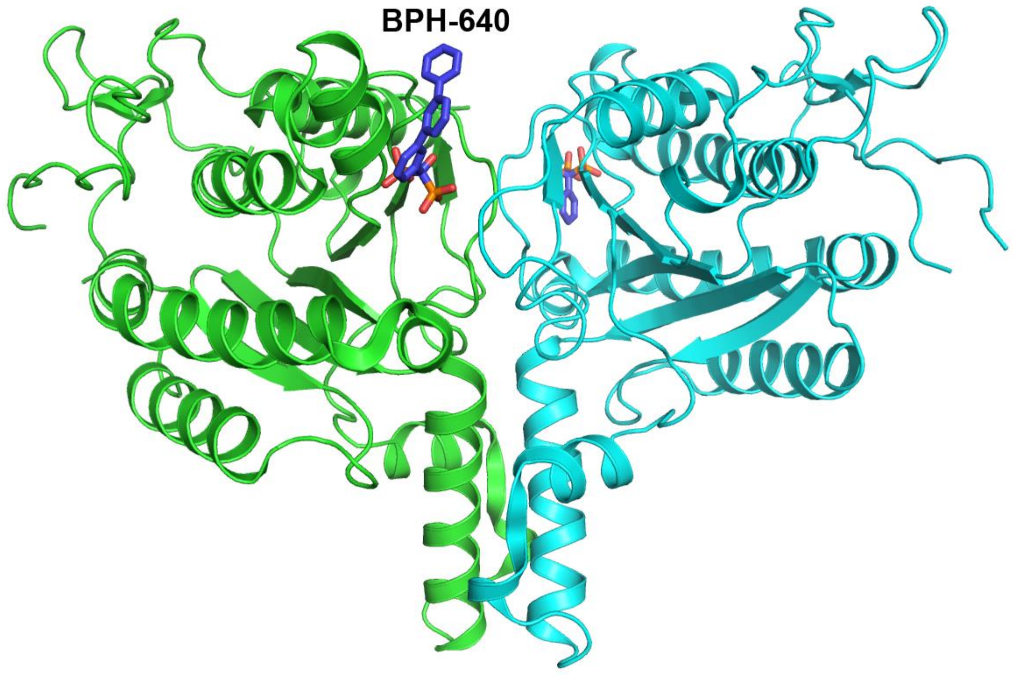

(*a*)

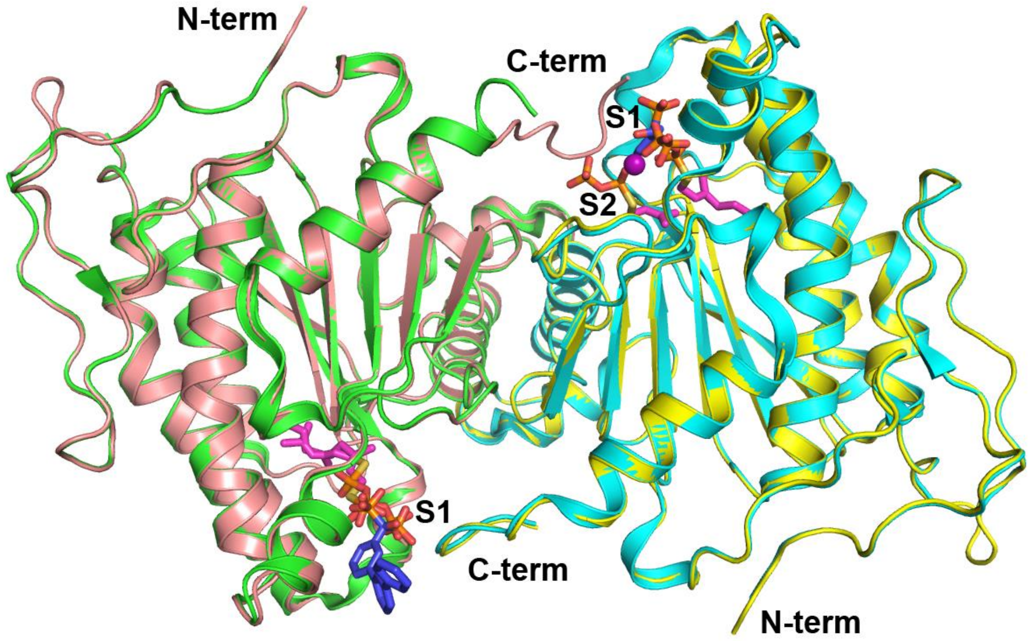

(*b*)

Figure S3 The bound ligands to MtDPPS in PDB 4onc, colored purple (*a*); and a comparison with those in this study, colored magenta (*b*). The latter protein is colored pink and yellow with the N and C

terminus of each chain indicated as well as the S1 and S2 site. For clarity, the view of (*b*) is from the top of (*a*), by a rotation of about 90°.

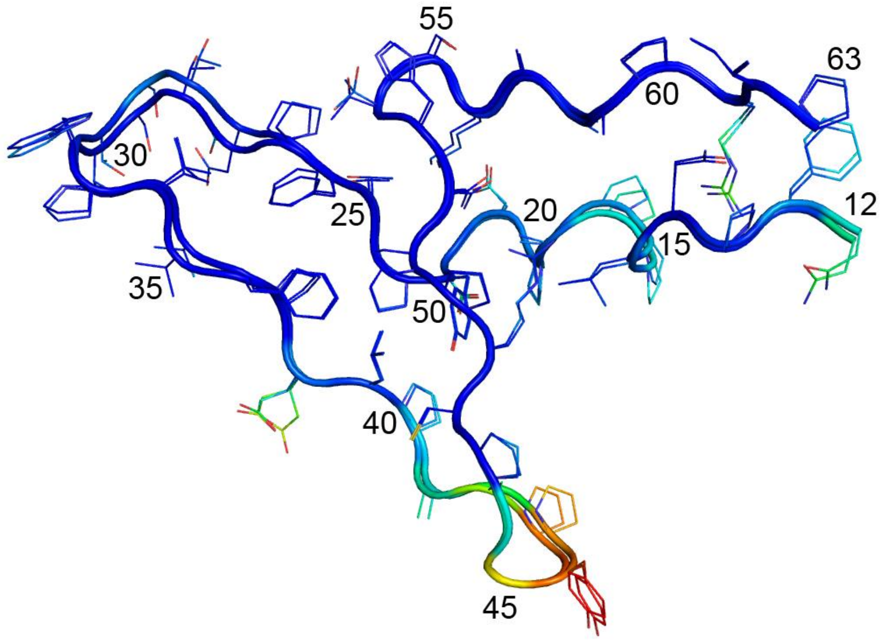

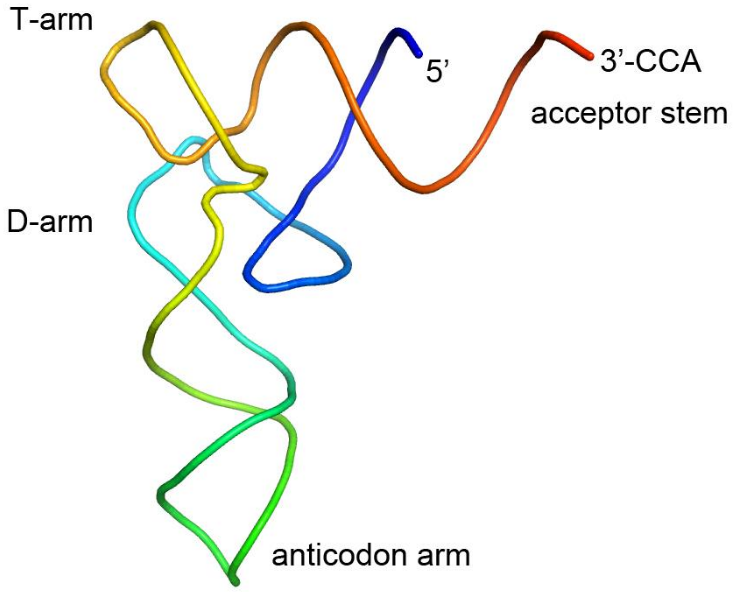

Figure S4 The unique N-terminal module of *Mt*DPPS (top), whose fold is reminiscent of a t-RNA (bottom). The tracing of *Mt*DPPS is colored according to temperature factors from blue (the coolest) to

red (the hottest). The tracing of tRNA may need a reversion from 3’-end to the 5’-end for a better match.

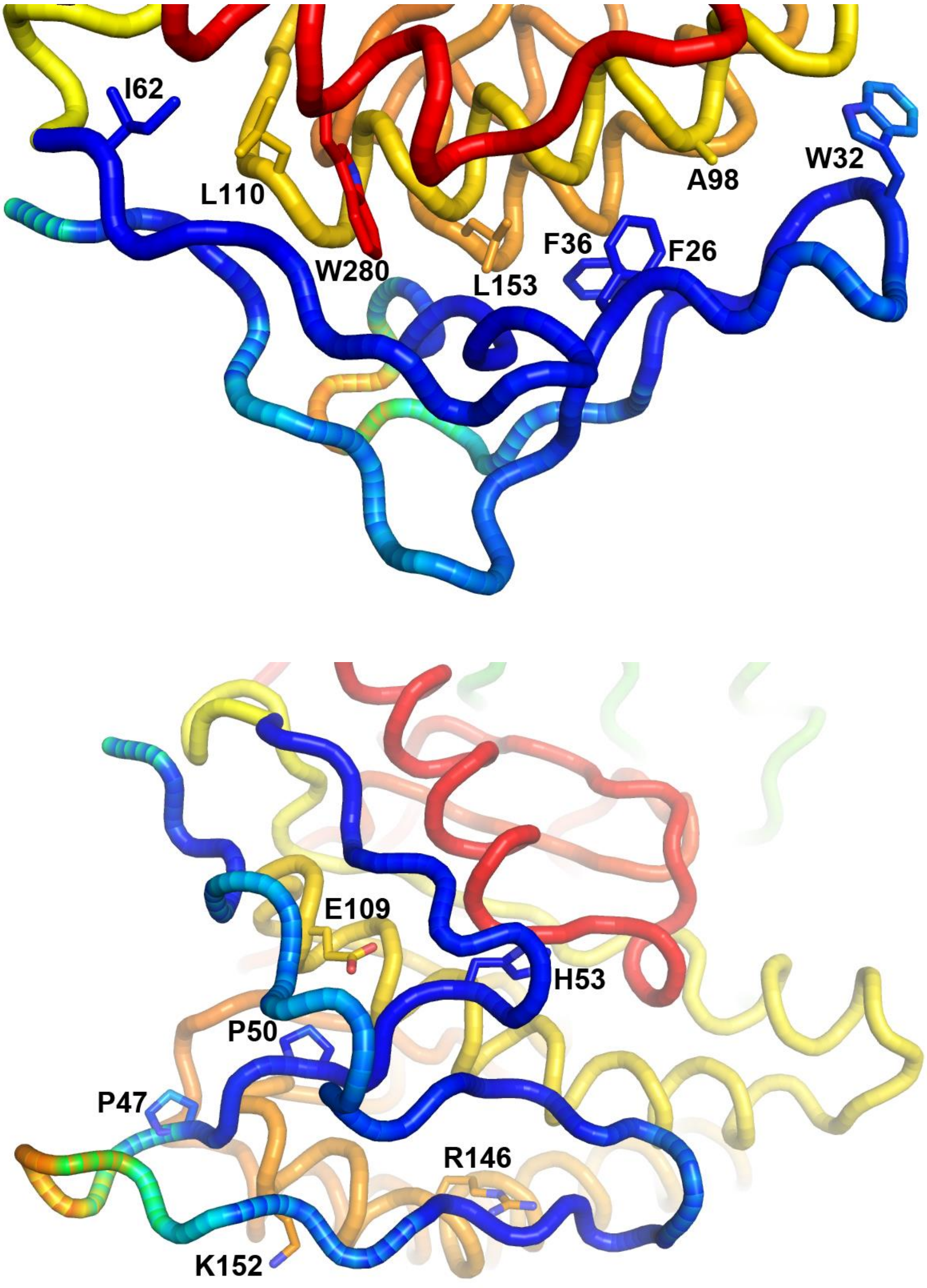

Figure S5 The interface of N-terminal module. The N-terminal module is colored according to the temperature factors as in Figure S4. The remaining part of *Mt*DPPS is colored from yellow to red. Major participating nonpolar amino acid side chains are shown as sticks in the upper panel, and so are the polar side chains in the lower panel.

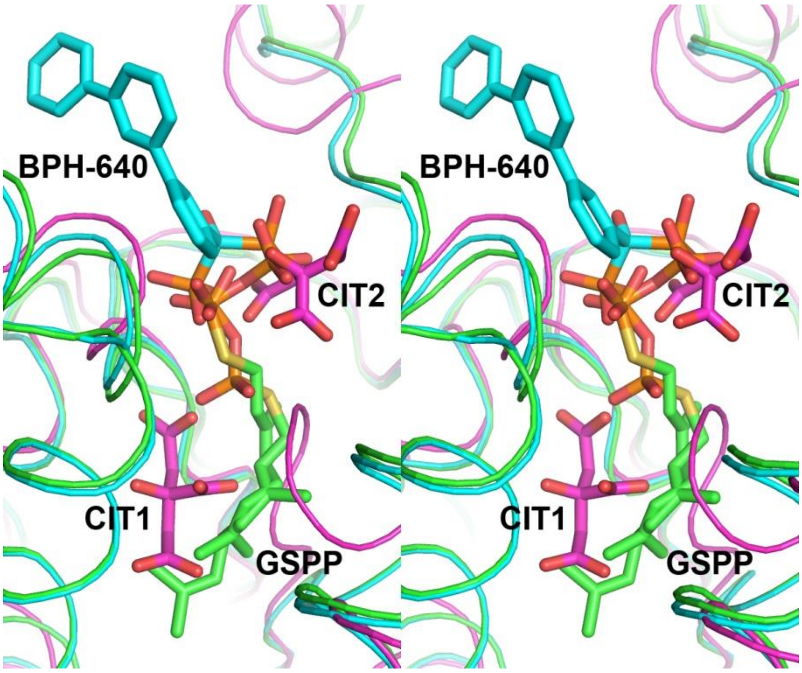

Figure S6 Structural comparison of the S1 ligands. In this stereo view of the S1 site, the proteins are shown as wire tracings colored green (for *Mt*DPPS in this study), cyan (*Mt*DPPS in PDB 4onc) and magenta (*Acinetobacter* UPPS in PDB 6acs). The bound ligands GSPP, BPH-640, and citrate (CIT1 and CIT2) are shown as stick models with the respective colors. The GSPP shown here has two alternate conformations.
